# Unveiling school community perceptions of nutrition-friendly school initiatives in preschool settings in rural Sindh province, Pakistan: an exploratory study

**DOI:** 10.3389/fpubh.2024.1379229

**Published:** 2024-05-15

**Authors:** Amir Ali Samnani, Rozina Karmaliani, Rozina Nuruddin, Pammla Petrucka, Sajid Bashir Soofi

**Affiliations:** ^1^Department of Community Health Sciences, Aga Khan University, Karachi, Pakistan; ^2^Department of Brain & Mind Institute, Aga Khan University, Karachi, Pakistan; ^3^School of Nursing and Midwifery, Aga Khan University, Karachi, Pakistan; ^4^Clinic Place Saskatchewan, College of Nursing, University of Saskatchewan, Saskatoon, SK, Canada; ^5^Centre of Excellence in Women and Child Health, Aga Khan University, Karachi, Pakistan; ^6^Department of Pediatrics and Child Health, Aga Khan University, Karachi, Pakistan

**Keywords:** early child development, malnutrition, nutrition interventions, nutrition friendly school initiative, preschool

## Abstract

**Introduction:**

In 1995, the World Health Organization launched its Global School Health Initiative to expand the Health Promoting School (HPS). The objective of this study was to explore the perception of the school community in implementing nutrition-friendly school initiatives in preschool settings.

**Methods:**

This paper delineates the exploratory phase of a mixed-method study, which broadly aims to assess the adoption of the Nutrition Friendly School Initiative (NFSI) through a preschool preparedness intervention package in rural Sindh province, Pakistan. The study sites include three campuses of the Aga Khan School (Mirpur Sakro, Junior Campus Vur, and a community-based school in Sujawal). Participants were selected purposively from these campuses and constituted a committee named ‘school community,’ which was responsible for implementing all activities outlined in the intervention package. Data was gathered through in-depth interviews and consultative meeting with the school community. Thematic analysis was employed for data analysis.

**Results:**

The analysis established five major themes that represent the participants’ perception of school-based nutrition interventions in preschool settings. These five themes include (i) Challenges in health and nutrition for pre-school age children, (ii) Clarity in roles and responsibilities for school-based nutrition intervention, (iii) Advancing school-based nutrition activities and interventions, (iv) Recognizing resources requirements, (v) Opportunities and challenges for the way forward in school-based nutrition. Findings also suggest sustainability and scalability measures that include the aligning School Nutrition Policy with the school handbook, ways to engage with parents, a nutrition theme corner, the inclusion of a parenting component in the nutrition manual, and capacity building of the school community.

**Conclusion:**

Qualitative findings have guided the refinement of the intervention package, proposing additional measures for sustainability and scalability across AKES, P. The school community is hopeful that the implementation of the refined intervention package will enhance preschool preparedness toward achieving nutrition-friendly school checklist items. This study holds strong potential for replication in a public school setting and presents an opportunity to launch a school accreditation program to certify schools as Nutrition-friendly.

## Introduction

Child malnutrition is a serious public health issue. According to the Joint Child Malnutrition Report [UNICEF, WHO, and World Bank Group (1)], approximately 148 million under-five children (22.3%) are affected by stunting (short height-for-age) while 45 million (6.8%) are wasted (low weight-for-height), Globally. More than two-thirds (70.23%) of all wasted under-five children lived in Asia (1).

In the realm of malnutrition, South Asia bears the greatest global burden, with statistics revealing that the region encompasses 52% of stunting cases, 70% of wasting instances, and 48% of overweight occurrences, a phenomenon often referred to as the “South Asian enigma” (1). Predominantly, over half of malnourished children in South Asia are concentrated in Pakistan, Bangladesh, and India (2).

Pakistan is facing a triple burden of malnutrition where 40.2, 17.7, and 29.9%, are found to be stunted, wasted, or underweight (low weight-for-age) while 9.5% are overweight (2). Additionally, more than half (53.7%) of Pakistani children are anemic and 28.6% have iron deficiency anemia. Deficiencies of other micronutrients – such as zinc (18.6%), Vitamin A (51.5%), and Vitamin D (62.7%) are also common (2). The prevalence of stunting improved from 1965 (48%) to 1994 (36.3%) but worsened from 2001 (41.6%) to 2011 (43.7%). As of 2018, it remains high at 40.2%, with an average annual reduction rate of 0.5%, insufficient to notably reduce stunting in Pakistan. Similarly, wasting has increased steadily since 1997, rising from 8.6% in 1997 to 17.7% in 2018. Sindh province has the highest rates of wasting and underweight nationwide (3).

The Government of Pakistan (GoP) has demonstrated a significant commitment to combat malnutrition through various initiatives. The establishment of the National Nutrition Forum (NNF) at the Planning Commission serves as a high-level nutrition governance forum aimed at providing leadership in harmonizing nutrition programs, coordinating efforts, and planning policies nationwide. Furthermore, the Federal Government has recently launched the Pakistan Nutrition Initiative (PANI), a comprehensive national program targeting malnutrition in 36 high-impact districts across the country. Recommendations have also been put forward for the establishment of a national nutrition dashboard to monitor key indicators, highlighting the importance of Early Childhood Development (ECD) in fostering resilient and capable individuals by nurturing cognitive, social, emotional, and physical growth (4).

ECD is multidimensional and involves an ordered progression of motor, cognitive, language, socio-emotional, and regulatory skills, and capacities across the first few years of life (5). The impacts of early life events, encompassing nutritional deficiencies and toxic stress, can vary in their effects on developing brain regions, contingent upon the timing, dosage, and duration of such occurrences. Generally, the earlier the adversity occurs, the greater the likelihood that the hippocampus will experience more pronounced effects compared to the prefrontal cortex (6).

There are clear implications of the timing of stunting and how it affects our conceptualization of the nexus between health, nutrition, and early child development. As with linear growth, the early years of a child’s life are critical for cognitive and socio-emotional development. Early childhood development (ECD) interventions delivered to children aged <5 years have been shown to have substantial and sustained impacts on long-term cognition and neuro-developmental outcomes (7).

In 1995, the World Health Organization (WHO) launched its Global School Health Initiative (GSHI) to expand the Health Promoting School (HPS) approach globally (8). This initiative was guided by the Ottawa Charter of Health Promotion, promulgated in 1986 (8). It is designed to improve the health of students and the school community through schools at all levels (9).

As an effort to halt the progression of chronic, non-communicable diseases, the World Health Organization (WHO) called upon countries to adopt school policies and programs that promote a healthy diet and physical activity, under a school policy framework (10) followed by the Nutrition-Friendly Schools Initiative (NFSI) which was launched by the WHO, following expert consultations on childhood obesity held in Kobe, Japan in 2005 (11). The benefits of adopting NFSI include, but are not limited to (i) helping schools to build an enabling environment for promoting the overall health and nutritional well-being of children; (ii) strengthening the capacity of schools in addressing the health and nutritional problems of the children, their families, and communities; and (iii) promote networking among the school community, including school personnel, students, parents and education authorities in alleviating the burden of malnutrition (11).

In this context, schools represent a prominent setting to protect, promote, and support adequate nutrition in children, thus offering a chance to encourage healthy eating habits from childhood onwards (12). School-based nutrition interventions can address various components, including setting standards for the food, meals, and beverages served in the school, as well as other actions aimed at improving the school environment, such as banning vending machines and regulating food advertisements (13). Additionally, School nutrition programs also comprise a curriculum on nutrition and healthy diets, offer school health and nutrition services, and extended opportunities to engage parents and the communities for the improvement of dietary practices amongst school children (13).

Recognizing malnutrition’s significant impact on hindering optimal child development and subsequent national progress, there is a clear rationale for integrating nutrition interventions into preschool environments. The conceptually outlined pillars of the Nutrition Friendly School Initiative (NFSI), encompassing Nutrition-Friendly Policy, awareness building, capacity development, manual development, support for school environment, and nutrition and health services, aim to prevent various forms of malnutrition and provide a framework for self-assessment (baseline & end line) of preschool readiness for the subsequent quantitative phase of the exploratory-sequential mixed-method design. Thus, the qualitative phase aims to explore the perceptions of the school community regarding the implementation of nutrition-friendly school initiatives in rural preschool settings in Sindh province, Pakistan. This exploration seeks to ascertain the intent to deliver, the feasibility of adoption, and the required resources for sustaining nutrition-friendly initiatives. The qualitative phase guided the understanding of the implementation process and further refinement of the intervention package in the sequential quantitative component of the study.

**Figure 1:**
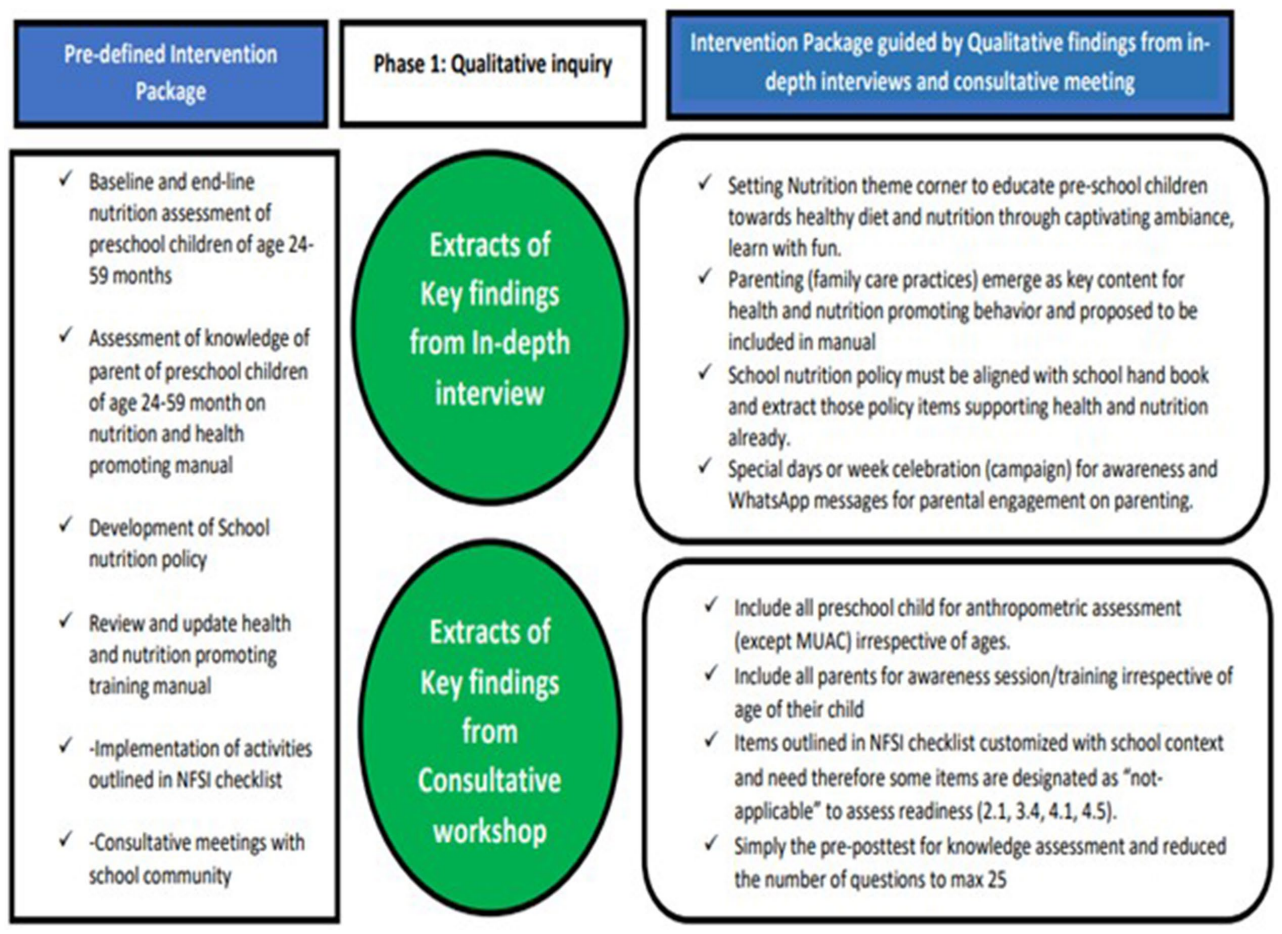
Refined intervention package guided by qualitative findings from IDIs and a consultative meeting with the school community.

The action framework was developed, containing five pillars of NFSI and proposed strategies and anticipated outcomes through this initiative (Figure 1). These five pillars of NFSI were used to assess baseline readiness. This action framework will be modified in the implementation phase based on findings obtained from this qualitative inquiry.

## Methods and materials

### Study design and its rationale

This paper elucidates the exploratory phase within the framework of an “exploratory sequential mixed method design.” The exploratory sequential approach is one of several mixed methods study designs that integrate qualitative (qual) and quantitative (quan) data collection and analysis across sequential phases (14). It initiates with qualitative data collection and analysis, preceding the quantitative phase where findings from the qualitative stage inform subsequent quantitative analyses.

Mixed-method research embodies a pragmatic worldview, amalgamating elements of both positivism and constructivism (14). Positivism, a philosophical approach, underscores the significance of empirical evidence and scientific methodologies in comprehending the world. In contrast, constructivism, a philosophical and educational theory, posits that individuals construct their understanding and knowledge of the world through their experiences and interactions, advocating for an active, internal learning process wherein learners construct meaning rather than passively absorbing knowledge from external sources (14).

The exploratory design facilitates participants in openly expressing their perspectives and allows for the researcher’s reflective engagement to provide contextual meaning to the data (14). The study adhered to an interpretive paradigm, In this paradigm, researchers seek to uncover the meanings and interpretations that people attach to their experiences, rather than focusing solely on objective facts (14). The chosen approach is grounded in the need to explore subjective viewpoints on the necessity and significance of such initiatives at the preschool level. This exploration aimed to determine the feasibility and status of resources to adopt and sustain this initiative. This exploratory phase has guided the implementation according to the five pillars of NFSI (Figure 1) and the subsequent quantitative phase of the mixed-method study (reported separately).

### Study participants

A school community was constituted with the support of school management that has representation from each campus. A school community (SC) comprised of representatives from the school management committee (SMC), parent-teacher committee (PTC), head teacher of the campuses, and eligible preschool teachers.

To be eligible for participation, school community members were required to have a minimum affiliation of at least 6 months with the school, be available during the study’s implementation phase, and receive a nomination from the school principal to ensure that only those teachers fulfilling the criteria were selected and commit to continued involvement with the preschool for the next 6 months. The school community led the NFSI activities due to the centralized administrative structure, with a single management overseeing all three campuses.

### Study setting and sites

This study was conducted at three community-based preschool sites of Aga Khan School campuses located in Thatta (Mirpur Sakro & Vur) and Sujawal districts of Sindh province. These rural communities face significant challenges regarding access to basic health and nutrition facilities. These school campuses fall under the management of Aga Khan Education Service - Pakistan (AKESP). The selection of study sites was done in collaboration with the head of programs. AKESP operates as a private entity within the Aga Khan Development Network (AKDN) and currently oversees 153 schools and five hostels throughout Pakistan (15), providing quality education spanning from pre-primary to higher secondary school levels. The rationale behind selecting private schools is the feasibility, flexibility, and likely readiness of school management to mobilize resources to implement, adopt, and sustain the intervention as described in NFSI. These schools have a structured pre-primary curriculum run by teachers trained in early childhood development which is not available in current public school’s set-up.

### Data collection methods and process

Qualitative data collection involved conducting in-depth interviews (IDIs) and a consultative meeting with the school community at the project’s outset. Five IDIs were conducted: one with the school principal, one with the headmaster of each selected site, and three paired interviews at each site involving a teacher and a school management representative. These interviews aimed to gather technical and operational insights and were conducted in person at the respective preschool sites.

Interviews were audio-recorded to ensure accuracy, with note-taking capturing essential details, including verbal and non-verbal cues. Each interview, lasting 45–60 min, followed by signed informed consent and accommodated participants’ availability, conducted in English or Urdu as per preference.

A self-developed structured IDI guide (see Annexure 2) with open-ended questions facilitated comprehensive discussions. IDIs aimed to capture subjective viewpoints relevant to exploring implementation processes and shaping interventions. Additionally, a four-hour consultative workshop during the preparatory phase engaged the school community in discussing specific aspects such as policy outlines, nutrition and health manuals, and operationalizing nutrition screening. These discussions have also contributed to the qualitative analysis.

### Data analysis

The qualitative data collected in this study underwent thematic analysis, a methodological approach that emphasizes the identification, analysis, and interpretation of patterns of meaning within the dataset (14). Thematic analysis involves systematically examining the data to identify recurring themes, which are then analyzed and reported to provide insights into the underlying phenomena (14). Each interview conducted as part of the study was audio recorded and transcribed verbatim. These transcripts were then imported into a Microsoft Word file for analysis.

Upon thorough examination of the transcribed data, the process of coding was initiated. Coding entails the systematic reduction of the data into meaningful segments, to which labels or codes are assigned (16). This process was conducted manually, involving multiple reviews of the transcripts to identify and label segments of significance. Subsequently, these codes were organized into sub-categories, categories, and overarching themes, allowing for a comprehensive understanding of the data.

Additionally, the key points discussed during the consultative inception meeting were documented separately. These discussion points were integrated into the analysis process, providing further insights that contributed to the customization of the intervention package based on the main findings of the study.

### Ethical considerations

The study protocol was approved by the Ethical Review Committee (ERC) of Aga Khan University – AKU Ref #2021–6,622-20068 (Annexure 3). In addition, the following ethical measures were considered in the conduct of this study:

- *Informed consent:* Verbal and signed consent was obtained from study participants (school community), providing clear and concise details about the study purpose, risks, and benefits of participation (Annexure 4). Although the consent was taken in English, the content and information on the consent form were explained in Urdu.
- *Confidentiality and anonymity*: Participants were informed about the audio recording of interviews before the initiation of interviews and were assured of confidentiality during and after the study. The recordings were securely transferred to a separate laptop and permanently deleted from the mobile device.
- *Autonomy:* Participants were informed that they could withdraw from the study at any time without facing any penalty. They were also informed that they had the right to refuse to answer any questions if they felt uncomfortable doing so.
- *Incentives:* Participants were informed through the consent form that there were no direct incentives or compensation provided to participants during the study. At the end of the study, participants received a certificate of appreciation and a souvenir (for each site) as a token of remembrance.

### Study rigor

The qualitative approach’s rigor was maintained by ensuring the trustworthiness of the research through credibility, dependability, conformability, and transferability (14). The credibility of the study findings was ensured through triangulation, combining findings from in-depth interviews and the consultative inception meeting. Furthermore, triangulation was achieved by interviewing a diverse group of participants representing the school community (14). Dependability was ensured by maintaining an audit trail that documented every step of the data collection process. Additionally, each participant reviewed and signed their transcripts post-compilation, and counter-signed by the head teacher. Interviews were conducted until data saturation was reached, ensuring no new themes emerged. This has further enhanced the credibility and dependability of the findings. To ensure the conformability of the study, the construction of codes, subcategories, and categories was extensively discussed with team members to reach a consensus (14). Additionally, the researcher double-checked unclear findings with participants after each interview. While generalizability is not a primary goal in qualitative studies, transferability is desirable. This was achieved by offering a thick description of the study context and the phenomenon of interest, enabling conclusions to be drawn from other settings (14).

## Results

A total of five (*n* = 5) IDIs were conducted with nine participants from the school community. The conceptual framework resulting from this study comprised five key themes, encapsulating 15 sub-themes and 35 categories. The thematic analysis of the findings is presented in Table 1.

**Table 1:** Thematic analysis of qualitative findings.

Sub-themes	Categories	Codes
Theme 1; challenges in the health and nutrition of preschool-age children		
Health and nutrition issues among preschool children aged 24–59 months	Identifying health issues	Frequent illness (2), vision issues (1) general weakness (4) vomiting (1), diarrhea (1), seasonal/viral illnesses (1), low immunity (1), feeling sleepy (1)
	Identifying nutrition issues	malnutrition (1), anemia (1), short height for age (2)
	Identifying behavioral and related issues	Increase absenteeism because of illness (1), children coming to school without breakfast (2), attention deficit (1), poor hygiene issues (1), increase junk consumption (2) less preference for home-made food (1)
Activities, Measures, and mechanisms are in place (or in practice) in school for optimal preschool children’s health and nutrition status	Promoting physical activity	Sports (1), yoga (1), exercise (2), meditation (1)
	Applying health and hygiene measures	Invoke junk food restrictions (3), apply COVID measures (temp check, hand sanitizer) (1), conduct annual/*ad-hoc* health assessment (3), promote hygiene practices (2), encourage home-made foods (1)
	Employing a check and balance mechanism	Check and balance over lunch box (3) and hand washing before and after having lunch (2)
Knowledge of parents/caregivers regarding healthy diet and nutrition status for preschool children	Parents/caregivers have minimal knowledge	Parents are well aware (2), parents have superficial/ insufficient knowledge (about healthy diet (1), nutrition status of a child) (2)
	Parents/caregivers lack knowledge	Parents are unwitting (1), lack knowledge/ awareness (2)
	Other related aspects	Compliance issue (1), incapability to manage or adopt good practices (1), parents do not have time to look after their child/ren (2), need to sensitize parents (2) (on child development, healthy upbringing, milestone tracking), parenting issues (2)
*Theme 2: Clarity in roles and responsibilities for school-based nutrition intervention [102 responses]*		
Roles and responsibilities as implementors of nutrition initiatives	Parents as stakeholders	Overall care of child (3), nurturing of child (2), taking care of hygiene (2), healthy lunch/ diet (home-based) (2), child development (1), quality parenting (3), adopting messages and guidance provided by school (1), nutritional care (2) and safe drinking water (1), ensure quality sleep of child (1), monitor child routine (1), take interest in school activities (1) restrict junk food (1), discourage giving lavish food (1), develop a taste preference for a child (1), meal modification to it more palatable (1), parental attention towards child (1)
	Teachers and school committee as stakeholders	Remain vigilant (1), provide a proper menu for lunch boxes (1), encourage parents to adopt schools’ initiatives (1), communicate with parents (2), the bridge between parents and school (3), and awareness raising (2), sharing circular to keep parents informed (1), adopting this initiative (2), contributing towards school nutrition policy (1), guide the child (1), work on sustainability of this initiative (1), monitoring compliance (3)
	Role of Children as Stakeholders	Direct beneficiaries (1), main recipient (1)
	Others (think tanks and policymakers)	Work to sustain such initiatives (1), work on nutrition policy, and make this happen (1)
Role of school/ management in improving the nutrition status of children	Engaging with parents	Awareness raising sessions with parents; including hygiene (1), benefits of homemade lunch (1), health session (1), motivate parents (1)
	Employing a check and balance mechanism	Monitoring (1) and compliance (1) for lunch boxes and proper hygiene
	Promoting an enabling environment for health and nutrition	physical activity (4) exercise (1), yoga (1), sports (1), events (1) handwashing (1), discouraging/restricting junk food (2), school-based regular health checkups/ nutrition screening (2), special days celebration (2), identifying medical issues/ conditions (1), seeking expert opinion from AKU, AKHES, P) (1), setting up referral mechanism (1), encourage home-made lunches (2), healthy food sharing (1), nutrition policy for school (2) develop a structured food list (1) (emphasis on ‘to do rather than ‘not to do’)
Measures to ensure an enabling environment for improved nutrition	Improving engagement with parents	Awareness session with parents (4) - (on hygiene, healthy lunch, parenting, and child care); counseling of parents (2) - (for healthy food alternatives, practical alternatives to parents); engaging parents to discuss the health needs of their child (1); improving the communication gap regarding child health condition between teachers and parents through circulars and brochures (1), awareness raising campaigns (1), display of awareness banners (1), creating Whatsapp group (3), putting health and nutrition as separate agenda in parent-teacher meeting (1), continuous feedback on child progress (1), appreciation for parents as an incentive (1).
	Check and balance	Introducing a monitoring system (1), controlling junk food consumption (1), and establishing task-specific committees (1)
	Translating ideas and innovations into action (implementation measures)	Routine planner activities (1), prepare a list of healthy lunch box recipes (2) celebrating special days and weeks (3), school improvement plan (health and nutrition) (1), nutrition theme room/corner (2), learning with fun to introduce healthy eating (1), school nutrition assessment (2)
*Theme 3: Advancing school-based nutrition activities and interventions*		
Additional measures to promote health and nutrition	Sustain ongoing efforts in practice	Special days celebration (3), Hygiene awareness (1), Health inspection by the School Management committee (2), promoting Physical activities (1), & inspection of lunch boxes (healthy & homemade) (2)
	Additional measures for consideration	Activate certain task-specific committees (1), awareness on parenting (1) parental education and awareness program using a structured manual (2) developing the nutrition policy (3) translating policy into action (1) vitalization of a school health program (including nutrition assessment) (2)
Supporting parents to improve the health and nutrition status of children through the curriculum	Existing curriculum as a facilitator	School curriculum as facilitators (1), schools attempt to follow activities through a curriculum that responds to nutrition and health (1)
	Existing curriculum as a barrier	Lots of flows in the curriculum (1), curriculum not supporting nutrition (1) curriculum lacking health interaction (1), few activities are linked but not directly related to the curriculum (1)
Prioritizing interventions for future consideration	Prioritizing intervention at the system level (AKES, P)	Need for school nutrition policy for planning and guidance (1), making parents and children bound for screening (1), childhood vaccinations for admission eligibility and in checklist (1), routine school nutrition assessment (2), support the adoption of NFSI through this project (1)
	Prioritizing intervention at the school level (campus)	Linking our examples with healthy food items (1), food item distribution (1), control of junk food consumption (1), food days celebration (1), exposure visits to markets (1), diet-friendly educational poems and screening videos (1)
	Prioritizing interventions related to parental engagement	Awareness raising for parents (3), awareness on parenting (2), focusing on parental engagement (2), school-based competition (1), improving compliance measures (1), needs-based counseling and feedback to parents (1)
*Theme 4: Recognizing resources requirement*		
Capacity building of the school committee	Capacity building/orientation	Capacity building/ orientation for School committee (7) (on growth monitoring (3), child development (1), nutrition (2), parenting (1)) trained HR (1) to support the establishment of nutrition theme class (1)
Funding requirements	Funds/finances	Funds are required (8) for activities (1), refreshments, or food distribution (1) human resource (nurse, doctor, food technologist) (1), communication materials (1) requires school policy embedding funding for school nutrition programming: (1) need to incorporate in annual activity calendar for sustained funds (1), funds for setting nutrition corner/theme class (2)
Other resources/support	Others	Experts support from outside (2), technical support for setting nutrition corner (2), trained professional volunteers (1), additional HR (nurse) (2), IEC material (1)
*Theme 5: Opportunities and challenges for the way forward in school-based nutrition*		
Identifying the opportunities	Opportunities	The need is there (1), facilities and resources available for implementation (3), supportive leadership (2), an opportunity to have a healthy child (1), well-informed parents (1), a pro-nutrition school environment (1), school platform available (1), some good practices already exist so just need to build on that (1)
Identifying the challenges/gaps	Demand side (parents/caregivers)	Parental engagement and participation (response) (3), the acceptance level of parents differ (1)
	Supply-side (school/AKES, P)	Teacher engagement/time (2) until it becomes part of a policy, do not have nutrition policy (1), capacity gap in teachers and management to deliver nutrition initiatives (1) setting mechanism for routine assessment (1)
	External factors (COVID/Lockdown)	Unexpected school closure due to COVID (2), time shortage due to frequent off/lockdown, and frequent vaccine campaigns by the government (1)
Making the recommendations	System/ policy level (AKES, P)	Established nutrition assessment mechanism (1), include this initiative in annual school planner and budgeting (2), need for comprehensive school nutrition policy (2), setting up a nutrition corner/ theme class (2) include nutrition assessment for ECD class admission checklist (1), referral system/linkage need to be developed (1)
	School/implementation level	Orientation of the school committee on nutrition-friendly school initiative interventions for implementation (1), orientation of parents (3) (on positive parenting (2), nutrition (1)) training of teachers on nutrition assessment (1), Nutrition manual/package as a reference document and guide (1)

### Theme 1: challenges in health and nutrition for pre-school aged children

This theme outlined health and nutrition issues among preschool children emerged from three sub-themes

a *Health and Nutrition Issues among Pre-school Children aged 24–59 months:*

Health and Nutrition issues were grouped into health, behavioral, and nutrition categories. Regarding health issues, the identified concerns included frequent seasonal illnesses, general weakness, vomiting, diarrhea, and eyesight problems. Behavioral issues consisted of absenteeism, a preference for junk food, attending school without breakfast, attention deficit, and sleepiness in class. Students appeared untidy, as observed in their uniforms, nails, and overall hygiene. Nutrition-related issues included malnutrition, anemia, and short height for age.

*“… most children come to school without having breakfast, and teachers also arrive without breakfast most of the time. Children get tired while studying and lose attention…” [ECDT-CBS]*

b *Activities, Measures, and Mechanisms are in Place (or in Practice) in Schools for Optimal Preschool Children’s Health and Nutrition Status.*

The measures in-placed were categorized into health and hygiene, promoting physical activity, and implementing a check and balance mechanism. Health and hygiene measures include annual/*ad-hoc* health assessments, promoting COVID-related practices (hand sanitizer, temperature checks), and encouraging homemade foods over junk foods. Participants also emphasized promoting physical activity, such as exercise, yoga, meditation, and sports. Under the implementation of a check and balance mechanism, lunch boxes and hand hygiene before and after lunch were highlighted.

*“…in our school, we usually have a check-up once a year and we try to organize sports and other activities, but it did not happen last year due to COVID, however, children’s exercise sessions are conducted during assembly, but we can plan to set-up routine nutrition screening …” (HT-CBS)*

c *Knowledge of parents/caregivers regarding healthy diet and nutrition status*

Most responses in this category focused on “other related aspects” of childcare, encompassing parenting challenges due to time constraints, difficulty in managing, and the struggle to adopt good practices. There’s a need to raise awareness among parents about child development, health monitoring, and adherence to healthy practices. Additionally, four responses suggest that parents/caregivers possess limited knowledge regarding healthy diets and the nutrition status of their children.

*“We have mixed situation…parenting is a big issue. Some parents know but the problem lies in their behavior and practice. And some parents are not aware of their children’s diet and nutrition, so overall mixed situation.” [ECDT-CBS].*

### Theme 2: clarity in roles and responsibilities for school-based nutrition intervention

This theme has three sub-themes and ten categories.

a *Roles and Responsibilities as Implementers of Nutrition Initiatives*

Most respondents highlight the pivotal role of parents in implementing various aspects, including childcare, nurturing, and overall development, covering health, hygiene, diet, nutrition, quality sleep, and safe drinking water. Parents are also expected to engage in school activities, monitor routines, limit junk food, discourage lavish foods, shape the child’s taste preferences, and modify meals for better palatability.

*“Parents have a significant role, a big role. Since we are not a boarding school, parents play a major role. For example, we have a practice of bringing home-cooked food, but some parents include unhealthy items in the lunchbox, which defeats the purpose. The intention is to provide healthy homemade food instead of junk food. Some parents leave early for work, and if both parents are working, they may just put a cupcake or unhealthy snack in the lunchbox…” [P-RHO].*

Teachers and the school community also hold considerable responsibilities, including monitoring hygiene, offering proper lunch menus, and promoting healthy practices. They play a vital role in ensuring compliance, adapting, and sustaining initiatives, crafting a school nutrition policy, and maintaining constant communication with parents.

*“…structure food list item for lunch box needs to be prepared. We usually instruct parents on a “Not to-do list” so they do not send those items, we encourage home-made food…” [HT-MPS/Vur].*

Additionally, two respondents suggested that policymakers and school think tanks should collaborate on developing a sustainable school nutrition policy.

b *Role of School/ Management in Improving the Nutritional Status of Children*

The category underscores the importance of fostering a supportive health and nutrition environment. This involves initiatives like developing a school nutrition policy, conducting regular health checkups, and nutrition screening for preschool children. It also includes setting referral mechanisms [with Aga Khan University (AKU) and AKHS, P] for children with medical needs. In a similar context, emphasis was placed on raising awareness about healthy food choices through a structured menu and promoting physical activity and hygiene practices.

*“---being an educational institution, it is essential to identify medical issues and seek expert opinions or support from health agencies like AKHSP, and Aga Khan Maternal and Childcare Center (AKMCC). Proper referrals should be provided to them, and in some cases, firm action is necessary. At the same time parents should also be educated about the necessary steps to follow. Additionally, there is a need for school-based health screening with a proper setup…” [P-RHO].*

The second category focuses on engaging parents through awareness sessions on homemade lunches, health-related messages, and motivation for ongoing care. Lastly, the third sub-category highlights implementing a check and balance mechanism, primarily to monitor hygiene and lunch boxes.

c Measures to ensure an enabling environment for improved nutrition.

Measures were categorized into three categories. These categories include improving engagement with parents, translating ideas and innovations into action, and employing check and balance mechanisms. Engagement options with parents involve awareness sessions on hygiene, healthy lunches, child care, and parenting. It includes counseling on healthy food alternatives, practical tips, and discussions on the health needs of children. Respondents suggest additional methods like circulars, brochures, WhatsApp groups, banners, and highlighting health and nutrition in parent-teacher meetings. Recognition of positive parental efforts serves as an incentive.

*“…we can conduct awareness sessions with parents for 10–15 min. If children have a break at noon, parents usually arrive early, so it’s an opportunity for a parental session because parents are concerned about not giving junk food, so we can suggest alternatives to them…” [ECDT-Vur].*

Another category focuses on translating ideas into action, incorporating nutrition-oriented activities in the school planner, compiling healthy lunch box recipes, celebrating special events, and integrating nutrition assessments into the school improvement plan.

“…the school management should develop a school improvement plan to track health and nutrition progress” … [MT-MPS].

Finally, the last category stressed the importance of establishing a check and balance mechanism through task-specific committees to monitor lunch boxes and other health and nutrition aspects.

### Theme 3: advancing school-based nutrition activities and interventions

The theme has three sub-themes that emerged from seven categories.

a *Additional Measures to Promote Health and Nutrition.*

Activities encompass physical exercise, hygiene and diet awareness sessions, hygiene inspections, nutrition-focused celebrations on special days, and linking content with healthy snacks (e.g., associating colors with fruits). SMC conducts health inspections. Respondents propose sustaining the program and enhancing nutrition-friendly schools through task-specific committees, structured parenting education, nutrition policy development, translating policy into action, and revitalizing school health and nutrition assessments.

*“…School management committee can conduct health inspections and monitoring compliance…” [HT-CBS]*

b *Supporting Parents to Improved Health and Nutrition Status of Children through Curriculum.*

Participants drew attention to the flaws in the curriculum as it does not support nutrition and lacks the interaction of the health component. According to one response, few activities are linked to the existing curriculum at an indirect level. However, two respondents stated curriculum acts as a facilitator and there are activities that schools follow that respond to nutrition and health.

*“…as such, I see many flaws in it. I do not see much of a nutrition support curriculum, especially in subjects like language-based subjects, except for science or biology, where there might be one chapter on nutrition, but otherwise, I could not find any health-related content…” [P-RHO]*

c *Prioritizing Interventions for Future Consideration*

Systemically (as AKES, P), future priorities involve establishing a school nutrition policy, mandatory screening, and vaccination for school admission, supporting NFSI adoption. At the school level, priorities include controlling junk food, celebrating food days, promoting healthy food choices through market visits, setting a nutrition theme corner in the class, screening diet-friendly content, and distributing healthy snacks. However, the primary focus (n = 10) is on enhancing parental awareness and engagement. Suggestions include school competitions for better compliance and needs-based counseling and feedback for parents.

*“…take children to places like the market (exposure visit) so that they can learn about locally available food …or setting a corner displaying fruits and vegetable charts, plastic toys for vegetable and fruits so children can spend time and listen to a nutrition and health-related poems and stories and screening of diet-friendly content, once a week to promote nutrition awareness…” [HT-CBS].*

### Theme 4: recognizing resources requirement

This theme has three sub-themes.

a *Capacity Building of the School Committee.*

The emphasis was on having trained human resources (HR) and teachers to sustain the nutrition program. Teachers must be trained for routine growth assessment and monitoring as well as establishing class themes. There should be an orientation of the committee responsible for growth monitoring and sessions for parents and setting up the nutrition theme corner.

*“There will be a need for teachers’ capacity building, and some resources may be required in terms of trained human resources…” [MT-CBS]*

b *Funding requirements.*

The participants highlighted the need for funds to sustain the nutrition project. The funds are required for all in-school activities and resources outlined in the NFSI checklist and specialized human resources (nurse and food technologist). This funding must be incorporated into the annual budget and activity planner.

*“Yes once the policy is in place and capacity is established to set up a system, we will need some funds. Funds will also be required for certain personnel such as a food technologist, HR personnel, and a school health nurse. Additionally, resources will be needed to develop communication materials” [P-RHO]*

c *Others.*

Participants stressed the need for miscellaneous resources or *ad-hoc* support like any external expert as a speaker or trained professional volunteer. Moreover, other support also includes information technology (IT) support, information education, and communication (IEC) material, stationary, etc.

*“…we can also anticipate human resource need for implementation once the program is fully implemented (if thinking in a long run purpose). One program should be structured and piloted here then we may think of scale-up and in such case, (we can request/propose additional HR support) we can call a counselor from Karachi for counseling…” [HT-MPS/Vur].*

### Theme 5: opportunities and challenges for the way forward in school-based nutrition

This theme emerged from three sub-themes and six categories.

a *Opportunities:*

The majority have seen opportunities in terms of what they already possess like the availability of facilities and resources for implementation, supportive leadership, a pro-nutrition school environment, and already-in-place good practices. Another opportunity was described as having well-informed parents leading to improved health of the child(ren). A single response emphasized the existing need to work on a child’s nutritional status and the betterment of future generations as an opportunity.

*“We also have some good practices in practice, like routine checking of the child (lunch box, uniform, nail, hygiene, etc…”) [MT-MPS]*

b *Challenges/gaps.*

Respondents stated that until nutrition is embedded in the school policy, nobody will acknowledge the importance of nutrition. Furthermore, the school does not have any school nutrition policy to sustain such initiatives. On demand side (parents/caregiver) gaps include the difference in perceptions and acceptance of each parent on the sensitivity of this issue and acknowledged that to have all parents engage and participate in this new initiative would be difficult.

*“…we have many parents whose acceptance levels* var*y, such as in the case of polio, typhoid, MR campaign, and COVID. So, the acceptance level of children by parents may differ…” [P-RHO]*

c *Recommendations*

System-level recommendations include a comprehensive school nutrition policy, a mechanism for assessment, referral, and follow-up, and incorporating a nutrition assessment indicator in preschool admission forms. These policy-related activities should be integrated into annual school planning and budgeting. At the implementation level, programs should reflect a nutrition focus, with ideas such as a nutrition corner or themed class, orienting school committees on nutrition intervention, and ongoing progress assessments. Finally, a suggestion includes providing parents with a nutrition guidebook and schools with a nutrition manual for curriculum integration and reference.

*“…school required nutrition policy. The policy will streamline all the process…” [P-RHO].*

## Findings from consultative meeting with school community

A consultative meeting was carried out to discuss the implementation aspect of this initiative and the activities outlined in the intervention package. The following suggestions were proposed

- Anthropometric Assessment: It was proposed by the school community that anthropometric assessment (excluding MUAC) should consider all preschool children irrespective of eligibility criteria to ensure inclusiveness and fairness.
- Awareness Raising Session with Parents: It was initially decided that a session would be planned for parents of children 24–59 months, but the participants suggested including parents of all the preschool children; therefore, it also included parents of children above 59 months.
- Awareness Raising Campaign for Teachers and Parents: An awareness-raising session and campaigns for parents on health and nutrition through in-person sessions and WhatsApp groups.
- Development of Health and Nutrition Promoting Manual: Participants flagged that so much work on health and nutrition manuals has been done in the past and utilized in several ECD-based teacher training. It was therefore recommended not to develop a new manual but to build on those available and include the key family care practice messages as a cornerstone for positive parenting.

### Utilizing the qualitative findings to guide the intervention package for the quantitative phase

**Figure 2:**
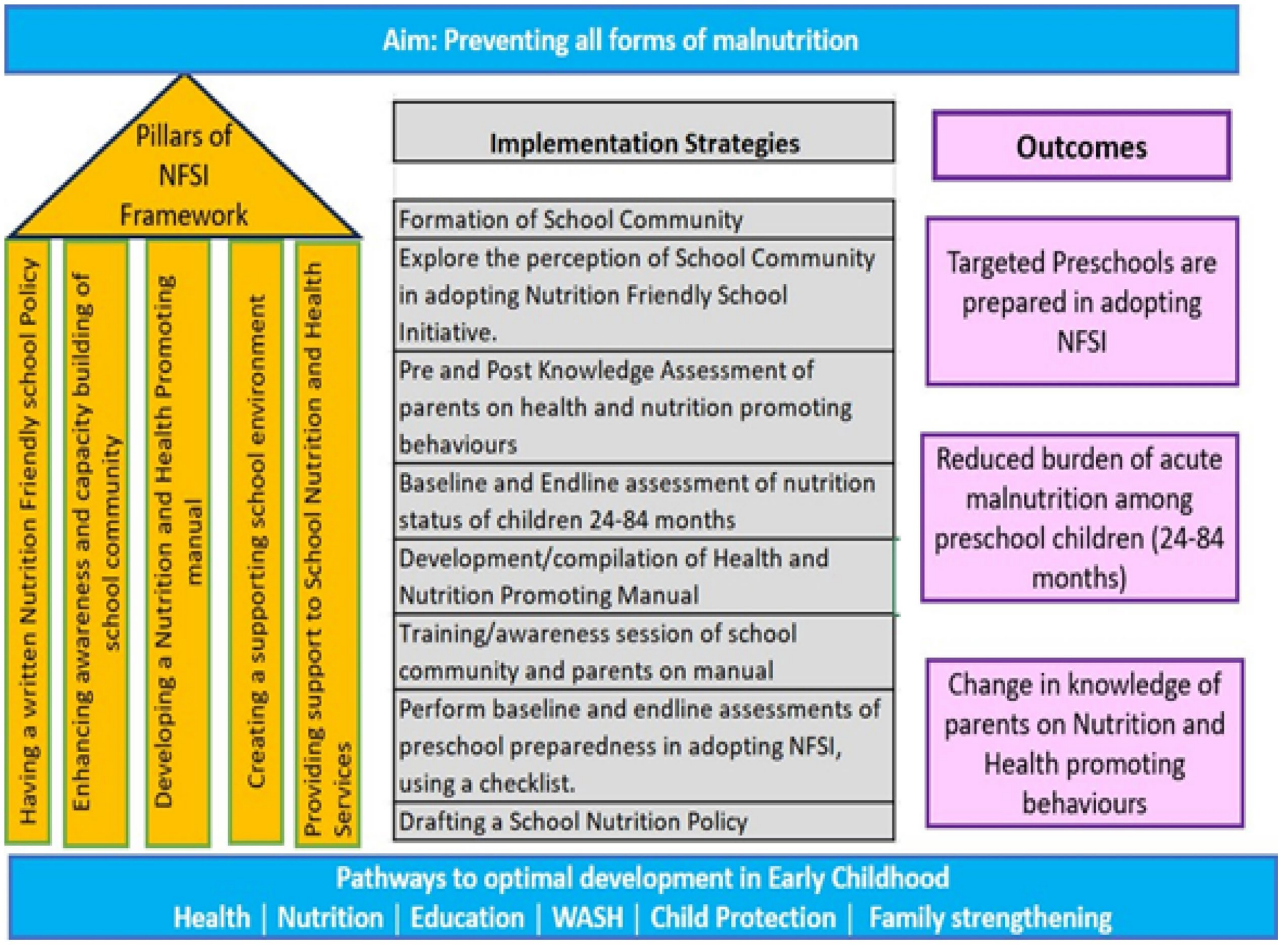
Action framework for implementing nutrition-friendly school initiative left SIDE: Five pillars from the framework adopted from the WHO framework for nutrition-friendly school initiative (11). The center of the framework represents the proposed implementation strategies after incorporating the responses from IDIs and the Consultative meeting. Right side: The anticipated outcomes.

Based on the analysis of findings obtained from in-depth interviews and suggestions proposed during a consultative meeting enabled the researcher to guide and upgrade the intervention package (refer to Figure 2).

## Discussion

This exploration has largely contributed to understanding the implementation process and informed the refinement of the intervention package. The key highlights from the exploratory phase include alignment of school nutrition policy with school handbook, inclusion of parenting component in nutrition manual, setting a mechanism for routine nutrition screening and referral linkages, engaging with parents/caregivers, and capacity building of school community and awareness raising for parents, creating an enabling environment through setting nutrition theme corner or classroom.

Study findings indicated that schools could become nutrition-friendly through the presence of a comprehensive school nutrition policy. The policy ensures the operational, financial, and technical adoption of activities. Once established, the school plans incorporate activities into the annual academic calendar, allocate budgetary resources, and ensure compliance. A systematic review synthesized international literature to assess primary school nutrition policies’ impact on food and beverage availability in schools. Systematic review evaluated the impact of primary school nutrition policies on the school food environment. Of 18 studies reviewed, 15 indicated positive effects, including increased availability of healthier foods and reduced availability of less healthy options. However, two (out of five studies) focused specifically on schools in low-income communities, reported a lack of financial resources as a barrier to implementing school nutrition policy in offering a wider selection of healthy foods and additional school resources as increasing the likelihood of offering healthy foods (17).

The health and nutrition promoting manual also emerged strongly during IDIs and consultative meeting. Parental awareness of nutrition-promoting behaviors and messages, and parenting competencies is crucial for meeting the health and dietary needs of children. A research study, part of the “Colorful Eating is Healthy Eating” program in Lublin’s kindergartens, in Poland, involved 11 institutions with 733 participating parents, and evaluated the effect of long-term nutrition education for 3- to 6-year-olds on parental nutrition knowledge. The program consists of six meetings (30–60 min each). Positive outcomes included a reduction in sweetened beverage consumption (*p* = 0.005), increased water intake (*p* = 0.001), and decreased sweets consumption was observed. The program improved parents’ knowledge of fiber sources and intake recommendations, emphasizing breakfast’s importance (18).

In the realm of integration of nutrition education within school curricula, this exploratory inquiry identified deficiencies in school curricula in addressing nutrition issues. A systematic review of School-Based Nutrition Programs in the Eastern Mediterranean Region scrutinized the inclusion of nutrition education in school curricula from the identified school-based programs (19). The topics covered within these nutrition education programs included lessons on healthy diet, the links between nutrition and health, healthy eating practices for the prevention of overweight and obesity, as well as healthy cooking practices (19). Many of these programs have also covered kindergartens along with schools. Seventeen countries out of 22, including Pakistan, have reported programs including nutrition education in their school curricula. In Pakistan, the emphasis has been on implementing corrective measures to tackle nutritional challenges that have adversely affected various facets of student well-being, including behavioral, cognitive, scholastic, and physical aspects, leading to elevated rates of morbidity and mortality and impeding socioeconomic progress (19). However, this integration was documented in only two provinces (Khyber Pakhtunkhwa and Balochistan), with no documented integration of nutrition education for other densely populated provinces. Thus, the imperative arises for the development and implementation of a unified national curriculum that holistically addresses nutrition with a prevention lens in both public and private settings schools.

The prevention aspect was also emphasized during discussions, particularly regarding school-based nutrition screening. The school community strongly suggested institutionalizing a nutrition screening mechanism that includes basic anthropometric assessments (height, weight, MUAC, BMI). Following suggestions received from the consultative meeting, the initial plan to assess children aged 24–59 months was modified to 24–84 months, ensuring the screening covers all preschool children. Additionally, establishing a referral and follow-up system is essential for providing need-based dietary counseling and linking with facilities for supplementary or therapeutic food to eligible children. These inputs were embedded within the intervention package. These findings are aligned with The Government of Pakistan supported the multidimensional “Tawana Pakistan Project” from September 2002 to June 2005, to address the undernutrition and low school enrolment of girls in primary school. At baseline and then every 6 months after that, weight and height were measured. Stunting, underweight, and wastage all saw declines of 6, 22, and 45%, respectively. This signified the potential of school nutrition programs to reduce the prevalence of different forms of malnutrition (20).

NFSI offers a framework for integrated interventions to enhance the health and nutrition of preschool and school-age children in a school setting. A study conducted in Gaza and the West Bank, Palestine, aimed at enhancing the nutrition well-being of school-age children through NFSI showed that the implementation of a context-specific intervention package elevated school nutrition to a national priority and increased the commitment of Palestinian authorities to lay the groundwork for future scalable interventions (21).

The school community is enthusiastic about implementing NFSI checklist actions, contingent on receiving necessary capacity building and support in creating an enabling environment. This support includes developing a school nutrition policy, and a nutrition manual, conducting periodic nutrition screening, parental engagement, establishing a nutrition-themed classroom or corner, and establishing referral linkages with community-based health services (AKHS, P & AKU). These measures are essential for ensuring sustainability and continuity of care. With supportive policies, schools can improve nutritional outcomes and successfully adopt this initiative.

This initiative not only improves nutrition and prevents malnutrition in preschoolers but also creates an enabling environment in schools for promoting overall health and well-being. It strengthens schools’ capacity to address health and nutrition issues within and beyond the classroom, allowing them to become accredited as ‘Nutrition-Friendly Schools’ and enhancing their reputation for investing effectively in the future generation (9).

## Strengths and limitations of the study

As for strengths, this study is pioneering and the first of its kind to explore NFSI implementation feasibility in rural Sindh. Among the study’s limitations is, an exclusive focus on private school settings, therefore, opinions, feasibility, acceptability, and adaptability in public schools may differ. Henceforth future studies in diverse settings, encompassing rural and urban areas as well as private and public schools, are warranted to comprehensively assess the real impact of this intervention. Secondly, this study does not capture perceptions from parents/caregivers due to operational issues and domestic engagements. However, two respondents representing the school community (interviewed) were also direct caregivers of children studying in preschool from the same campuses, hence providing a dual perspective (as a school community and caregiver).

## Conclusion

Qualitative findings have guided the refinement of the intervention package, proposing additional measures for sustainability and scalability across AKES, P., including the incorporation of parenting (key-family-care practices messages into the nutrition manual), aligning the school nutrition policy with the school handbook, and establishing a nutrition-themed corner to foster a nutrition-friendly environment, along with enhanced engagement with parents/caregivers. The school community is hopeful that the implementation of the refined intervention package will enhance preschool preparedness toward achieving nutrition-friendly school checklist items. This study holds strong potential for replication in a public school setting and presents an opportunity to launch a school accreditation program to certify schools as Nutrition-friendly.

## Data availability statement

The raw data supporting the conclusions of this article will be made available by the authors, without undue reservation.

## Ethics statement

The studies involving humans were approved by the Ethical Review Committee (ERC) of Aga Khan University – AKU Ref #2021-6622-20068 (Annexure 3). The studies were conducted in accordance with the local legislation and institutional requirements. The participants provided their written informed consent to participate in this study.

## Author contributions

AS: Conceptualization, Formal analysis, Investigation, Methodology, Project administration, Writing – original draft, Writing – review & editing. RK: Funding acquisition, Resources, Supervision, Writing – review & editing. RN: Writing – review & editing, Validation. PP: Methodology, Writing – review & editing. SS: Writing – review & editing.
