# Supplementary material for: Unveiling school community perceptions of nutrition-friendly school initiatives in preschool settings in rural Sindh province, Pakistan: an exploratory study
Source: Front Public Health. 2024 May 15;12:1379229. doi: 10.3389/fpubh.2024.1379229 (PMC11133731; doi:10.3389/fpubh.2024.1379229)
Supplement: Supplementary file 1 [file Data_Sheet_1.pdf]

Annexures

Annexure 1: Operational definitions of key terms

Early child development	ECD encompasses the physical, socio-emotional, cognitive, and motor development of children between 0-8 years of age
Nutrition Interventions	Nutrition interventions are policies, services, learning experiences, and other actions implemented by schools, individuals, or groups to make healthy nutrition a way of daily life
Malnutrition	Malnutrition includes overnutrition and nutritional deficiencies as well as undernutrition which impairs health, intellectual activity, adaptive behavior, education, productivity, and well-being, and can induce death
School Community	The school community comprised preschool teachers, a preschool principal, a representative from the preschool management committee, and a parent-teacher committee
Nutrition Friendly School Initiative	NFSI includes five components, which represent the conditions or criteria for schools to be considered nutrition-friendly:<br>- A written school policy on nutrition (also named Nutrition-Friendly Schools' policy)<br>- Awareness and capacity strengthening of the school community.<br>- Curriculum development and modification.<br>- Creation of a supportive school environment for optimal nutrition and health;<br>- School nutrition and health services
Preschool	A preschool, also known as a nursery school, pre-primary school, or play school, is an educational establishment or learning space offering early childhood education to children before they begin compulsory education at primary school.

Annexure 2: In-depth Interview Guide for Qualitative data collection

This guide is designed for In-depth interviews with School Community (School Headteacher, Principal, representative from Parent Teacher Committee, and ECD expert)

**Understanding health and nutrition in the context of a preschool setting**
What are the main health and nutrition issues among preschool children of age 24-59 months?
Does your school have any school-based Health and nutrition assessment program/s?
What measures/mechanisms are in place (or in practice) for the betterment of the health and nutrition status of preschool children?
Do you think parents have sufficient information/ knowledge regarding the healthy diet and nutrition status of their children?
**Understanding roles and responsibilities**
In your opinion, can school play their role in improving the health and nutrition status of preschool children?
What is the role of school/ management in improving the nutrition status of children?
What activities does the school/management consider to ensure an enabling environment for preschool children to attain improved health and nutrition status?
What is the role of parents in improving the nutrition status of children and how would your school have engaged parents to ensure it?
In your opinion how parents should be engaged with the school to support adopting nutrition initiatives in preschool settings?
**Understanding school-based nutrition activities and interventions**
Have you ever heard about school-based nutrition initiatives/Programs? If yes, please mention some of the key interventions preschool/ primary schools have adopted.
List down regular school activities that support the health and nutrition of preschool children.
In your opinion does the preschool curriculum support parents in improving the health and nutrition status of children?
In your opinion, what interventions school management should consider or focus on for preschool children?
**Understanding of resource requirement**
Who can be the main stakeholder/ person responsible for implementing such nutrition initiatives?
Does the school have the capacity to initiate or implement a nutrition-centered program?
Do you think adopting nutrition initiatives in preschool settings requires any funds to make it sustainable? Or it can be done through regular school activities
What additional resources school management would require to adopt school-based nutrition awareness?
Opportunities and challenges for a way forward and key recommendations

Annexure 3: ERC Approval letter

آغا خان یونیورسٹی
THE AGA KHAN UNIVERSITY

17-Jan-2022

Ms. Rozina Karmaliani
Department of School of Nursing and Midwifery
Aga Khan University
Karachi

Dear Ms. Rozina Karmaliani,

2021-6622-20068, Rozina Karmaliani: Assessment of Preschool Preparedness Intervention Package on Adoption of Nutrition Friendly School Initiative in Rural Sindh, Pakistan: An Exploratory Sequential Mixed Method Design.

Thank you for submitting your application for ethical approval regarding the above mentioned study.

Your study was reviewed and discussed in ERC meeting. There were no major ethical issues. The study was given an approval for a period of one year with effect from 17-Jan-2022. For further extension a request must be submitted along with the annual report.

List of document(s) approved with this submission.

Submission Document Name	Submission Document Date	Submission Document Version
CITI Course completion certificate_Dr Amir Ali Sammani (500940)		GCP
final version of questionnaire (prepost assessment) 02082021	05-Aug-2021	first
Anthropometric data recording sheet	05-Aug-2021	first
IDI guide for school community	05-Aug-2021	first
CITI Certificate-Dr RK	23-Aug-2020	valid
version 1 Socio Demographic Assessment for translation	13-Oct-2021	V1
V2 of questionnaire for parents (prepost assessment)	13-Oct-2021	V2
version 1 Socio Demographic Assessment	13-Oct-2021	V1
Urdu V2 consent form for IDI with School community	13-Oct-2021	V2
Urdu V2 consent form for parents	13-Oct-2021	V2
Affidavit of Translation	13-Oct-2021	V1
Dr Rozina Nuruddin CITI Certifcate	08-Jul-2021	V1
GCP Certificate - Dr. Sajid Soofi (1)	13-Oct-2021	V1
NIH ETHICS CERTIFICATE pammla	13-Oct-2021	V1
V3 consent form for parents	30-Oct-2021	version 3
version 3 consent form for IDI for School community	30-Oct-2021	version 3
Nutrition Friendly School Initiative Checklist	07-Dec-2021	1
V4 ERC revised submission version	08-Dec-2021	version 4
ERC Response sheet.docx 08122021	08-Dec-2021	latest submission

Any changes in the protocol or extension in the period of study should be notified to the Committee for prior approval. All informed consents should be retained for future reference.

Please ensure that all the national and institutional requirements are met.

Thank you.

Sincerely,

Dr Fyezah Jehan

Chairperson
Ethics Review Committee

Annexure 4: Inform consent form for IDI Participants

Title of study: Assessment of Preschool Preparedness Intervention Package on Adoption of Nutrition-Friendly School Initiative in Rural Sindh, Pakistan: An Exploratory Sequential Mixed Method Design

Principal Investigator: Dr Amir Ali Samnani **Committee members:** Dr. Rozina Karmaliani, Dr. Rozina Nuruddin, Dr. Sajid Soofi, Dr. Pammla Petruka

Introduction: Schools provide the most effective and efficient way to reach young children at influential stages in their lives when lifelong nutritional patterns are formed. Furthermore, schools may provide a setting to introduce nutrition information and technologies to the community and can lead the community in advocating for policies and services that promote good nutrition. Since you are eligible to participate in this study, I would like to invite you to join this research study.

Background: The burden of all forms of malnutrition is significantly high in Pakistan. Malnutrition refers to deficiencies, excesses, or imbalances in a person's intake of energy. According to Pakistan National Nutrition Survey (NNS-2018), the prevalence of stunting (short height for age) is 40%, whereas, wasting among children 6-59 months is on the rise from 8.6% in 1997 to 15.1% in 2011 and 17.7% in 2018 [3]. This is the highest rate of wasting in Pakistan's history since 1965. [3]. Besides this, over 40% of disadvantaged children under 5 years globally have neurodevelopmental problems resulting in deficits in social, emotional, and educational functioning into adulthood [4]. Early childhood development (ECD) interventions delivered to children aged less than 5 years have been clearly shown to have substantial and sustained impacts on long-term cognition and neurodevelopmental outcomes

Purpose of the Study: The purpose of this study is to pilot the implementation of Nutrition Friendly School Initiatives (NFSI) in preschool settings and to assess its outcomes in three major schools of AKESP, in Karachi, Pakistan by engaging school community (preschool teachers, ECD experts, Principal and representative from Parent teacher committees, etc.) Parents and preschool children, in adopting a nutrition-friendly school initiative through an iterative process.

Procedure: You are being requested to participate in this study, whereby you will be asked to share your perception regarding health and nutrition in the preschool setting, roles and responsibilities for child nourishment, school-based nutrition activities and interventions that support nutrition outcomes, resource requirements and opportunities and challenges for way forward. The purpose of IDI is to get an in-depth understanding of the context, need for such a program, and feasibility and willingness to implement. Based on the data collection findings, a Nutrition and health-promoting curriculum will be customized and the school community and parents will be trained on it. The interview questionnaire will take around 40-60 minutes. No identity-revealing information will be sought from you.

Possible Risks or Discomforts: There are no major risks associated with the study, however, if participants feel hesitant in responding to any question further clarity will be provided and they have an opportunity to skip any particular question to prevent any anxiety or stress. If needed, an interview will be interrupted till the participant feels comfortable.

Benefits: There are numerous benefits linked with participation in this study, Firstly, participants will get a chance to attend the training on nutrition and health promoting curriculum. Secondly, this pool of trained staff will receive a letter/certificate of appreciation from the section head of the school after the successful completion of this study. Moreover, these participants will also get a chance to attend the research dissemination event.

Financial Consideration: No financial compensations are involved for your participation in this study.

Voluntary Participation: Enrolment in this study is completely voluntary. You will have full right to withdraw from the study at any time and you may refuse to answer some or all questions if you do not feel comfortable. Refusal to participate will not in any way affect your child's enrollment or studies in school.

Confidentiality: The information shared by you will be kept confidential. Any identity-revealing information will not be mentioned in the questionnaire. All the data will be kept under a locked cabinet and the electronic files will be password protected. Access to study materials will be limited to the principal investigator and research team. The findings of the study will be shared or published in a journal without revealing your identity.

Available source of Information: If you have any queries or questions, you may contact the primary researcher: Dr. Amir Ali Samnani, Aga Khan University, Pakistan. Email: am_samnani@hotmail.com

Agreement to Participate: I have read and understood this consent form, and I agree to participate in this study. I understand that there are no major risks or benefits attached to this study. I also understand that the information that I provide, will be kept confidential. I voluntarily choose to participate, but I understand that my consent does not take away any legal rights. I further understand that nothing in this consent form is intended to replace my applicable federal, state, or local laws. I understand that a copy of this form will be provided to me.

Participant's Name and Signature: ______________________ Date: ________________

Principal investigator's Signature: _______________________ Date: ______________
